# Characterization of Equine Infectious Anemia Virus Long Terminal Repeat Quasispecies *In Vitro* and *In Vivo*

**DOI:** 10.1128/JVI.02150-17

**Published:** 2018-03-28

**Authors:** Xue-Feng Wang, Qiang Liu, Yu-Hong Wang, Shuai Wang, Jie Chen, Yue-Zhi Lin, Jian Ma, Jian-Hua Zhou, Xiaojun Wang

**Affiliations:** a State Key Laboratory of Veterinary Biotechnology, Harbin Veterinary Research Institute of the Chinese Academy of Agricultural Sciences, Harbin, China; b Department of Geriatrics and Gerontology, First Affiliated Hospital of Harbin Medical University, Harbin, China; Icahn School of Medicine at Mount Sinai

**Keywords:** EIAV, attenuated vaccine, LTR, molecular evolution, quasispecies

## Abstract

The equine infectious anemia virus (EIAV) attenuated vaccine was developed by long-term passaging of a field-isolated virulent strain in cross-species hosts, followed by successive cultivation in cells *in vitro*. To explore the molecular mechanism underlying the evolution of the EIAV attenuated vaccine, a systematic study focusing on long-terminal-repeat (LTR) variation in numerous virus strains ranging from virulent EIAV to attenuated EIAV was performed over time both *in vitro* and *in vivo*. Two hypervariable regions were identified within the U3 region in the enhancer region (EHR) and the negative regulatory element (NRE) and within the R region in the transcription start site (TSS) and the Tat-activating region (TAR). Among these sites, variation in the U3 region resulted in the formation of additional transcription factor binding sites; this variation of the *in vitro*-adapted strains was consistent with the loss of pathogenicity. Notably, the same LTR variation pattern was observed both *in vitro* and *in vivo*. Generally, the LTR variation in both the attenuated virus and the virulent strain fluctuated over time *in vivo*. Interestingly, the attenuated-virus-specific LTR variation was also detected in horses infected with the virulent strain, supporting the hypothesis that the evolution of an attenuated virus might have involved branching from EIAV quasispecies. This hypothesis was verified by phylogenetic analysis. The present systematic study examining the molecular evolution of attenuated EIAV from EIAV quasispecies may provide an informative model reflecting the evolution of similar lentiviruses.

**IMPORTANCE** The attenuated EIAV vaccine was the first lentiviral vaccine used to successfully control for equine infectious anemia in China. This vaccine provides an important reference for studying the relationship between EIAV gene variation and changes in biological characteristics. Importantly, the vaccine provides a model for the investigation of lentiviral quasispecies evolution. This study followed the “natural” development of the attenuated EIAV vaccine by use of a systematic analysis of LTR evolution *in vitro* and *in vivo*. The results revealed that the increase in LTR variation with passaging was accompanied by a decrease in virulence, which indicated that LTR variability might parallel the attenuation of virulence. Interestingly, the attenuated-virus-specific LTR variation was also detected in virulent-strain-infected horses, a finding consistent with those of previous investigations of *gp90* and *S2* evolution. Therefore, we present a hypothesis that the evolution of the attenuated virus may involve branching from EIAV quasispecies present *in vivo*.

## INTRODUCTION

Equine infectious anemia virus (EIAV) is a lentivirus characterized by the simplest genomic structure and mainly infects animals of the genus Equus. Distinct lentiviruses, including EIAV, human immunodeficiency virus type 1 (HIV-1), and simian immunodeficiency virus (SIV), have been reported to share many similarities in virus biology and host-to-virus immune mechanisms (1). In the 1970s, attenuated EIAV vaccines, including the donkey leukocyte-adapted strain (DLV121) and dermal-cell-adapted strain (FDDV13), were produced by sequential serial passage of a virulent field isolate of EIAV (LN40) *in vivo* in donkeys (DV117), in primary donkey monocyte-derived macrophages (dMDMs), and in fetal donkey dermal (FDD) cells. The prevalence of EIAV in China was controlled by nationwide utilization of EIAV DLV121 (2). Therefore, the attenuated EIAV vaccine system can serve as a model for studies of the nature and role of genomic sequence variation and can provide a useful lentiviral system for the natural immunologic control of lentiviruses.

The term “long terminal repeat” (LTR) refers to a type of common noncoding regions that are present in numerous retroviral genomes. LTRs, which are located at both ends of the proviral genome, consist of a U3 region, an R region, and a U5 region; they function as the viral promoter and contribute to viral replication and virulence (3, 4). The LTR regulates viral replication by interacting with the virus-encoded *trans*-activator protein (Tat) and a variety of host cell transcription factors. The expression profiles and levels of cellular transcription factors are determined by the cell type and the cellular immune environment, which regulate lentiviral replication (5). The ability of the virus to adapt to different target cell microenvironments is dependent on mutations in the LTR that generate different target cell transcription factor binding sites. A number of studies have reported that the LTR of EIAV exhibits a high mutation frequency after passaging of the virus in monocyte-derived macrophages (MDMs) or fibroblasts (dermal cells) *in vitro* (6–10). The LTR U3 region is the most commonly mutated region and contains multiple types of mutations, including substitutions, insertions, and deletions; this region can form target cell-specific transcription factor binding sites, such as PU.1 sites in macrophages (11, 12) and PEA-2 sites in dermal/endothelial cells (13, 14). In addition to the enhancer region (EHR), which has been reported by other investigators (7, 17), we identified mutation hot spots in the negative regulatory element (NRE), the transcription start site (TSS), and the *trans*-activation responsive (TAR) element in the R (repeated) region in previous studies (15, 16).

However, different results have been obtained for LTR variability during EIAV infection *in vivo*. Maury et al. found significant differences in LTRs within the same EIAV-infected individual, significantly higher LTR variation within individuals with clinical-onset EIA than within subclinical individuals, and large interanimal variation (17). While studies from the Montelaro laboratory showed that the LTR sequences of EIAV isolated from different organs of horses with infections spanning one and a half years were highly conserved with the inoculum, transient mutations and the transcription factor binding sites generated were also concentrated mainly in the EHR of the U3 region (18). By replacement of different LTRs in a series of infectious EIAV molecular clones, Payne et al. (3, 19) and other investigators (12) found that LTRs from different strains with mutations on the PU.1 binding site contributed to viral replication and virulence but that the LTR alone could not fully determine virulence.

Due to the inaccuracy and rapidity of RNA viral gene replication, viruses can develop a group of related variants termed quasispecies (20, 21). Many studies have shown that the presence of quasispecies is advantageous for viruses by facilitating their evasion of host immunity and adaptation to novel environments (22, 23). As with other classical attenuated vaccines, a series of virus strains differing in virulence and cell tropism was obtained during the derivation of the attenuated EIAV vaccines (DLV121 and FDDV13) in our laboratory (2). This vaccine system provides an important reference for studies of the relationship between EIAV gene mutations and changes in biological characteristics, as well as a model for the investigation of lentivirus quasispecies evolution. Although we previously reported several representative EIAV strains that were attenuated in cultivated dMDMs and FDD cells (16), the data were piecemeal. In this study, we systematically analyzed the evolution of the viral LTR during attenuation and during infection with virulent viruses and live vaccines. We discovered for the first time that the LTR had the same mutation pattern *in vitro* and *in vivo*, and we described an LTR quasispecies population in detail.

## RESULTS

**FIG 1:**
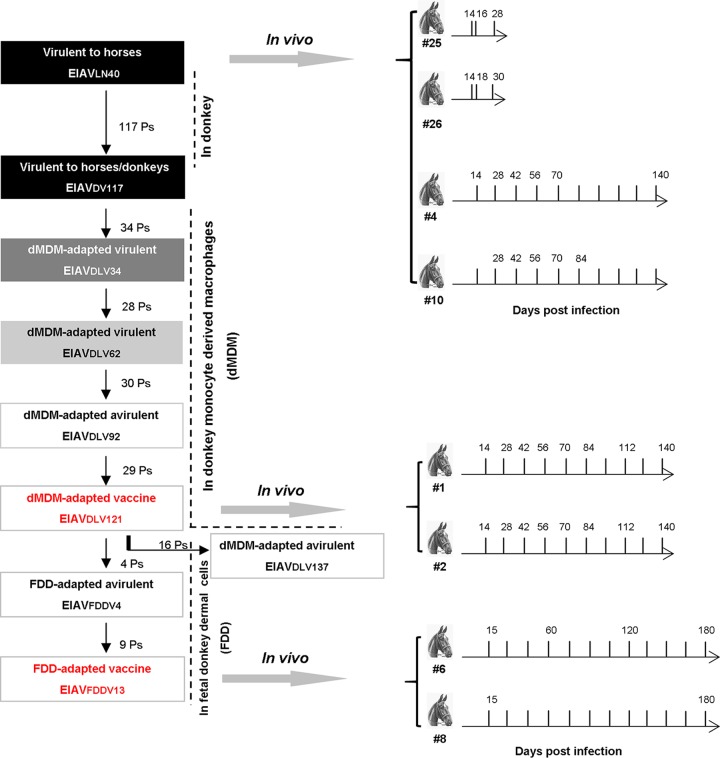
Schematic of the research strategy. LN40, an EIAV strain isolated from Liaoning, China, has undergone 16 consecutive passages in horses and is highly pathogenic to horses. After 117 consecutive passages (Ps) in donkeys, this virus was transformed into strain DV117, which is highly virulent in both horses and donkeys. The virulence of DV117 gradually decreased after consecutive passages in dMDMs *in vitro*. In this study, the proviral LTR sequences of the viruses after 34, 62, 92, 121, and 137 passages in dMDMs (strain DLV34 and its derivatives) and after 4 and 13 passages in FDD cells (strain FDDV4 and its derivatives) were sequenced. The gradual decrease in darkness from black to white indicates the reduction of virulence. The two live vaccine strains (DLV121 and FDDV13) are shown in red. The 34th generation (DLV34, virulent to horses), the 62nd generation (DLV62, virulent to horses), the 92nd generation (DLV92, avirulent but failing to induce protective immunity), the 121st generation (DLV121, avirulent and inducing protective immunity), and the 137th generation (DLV137, avirulent but having lost the ability to induce protective immunity) were sequenced. DLV121 was passaged for 13 generations in FDD cells to form the FDD-attenuated strain (FDDV13). We sequenced the LTRs of the 4th-generation DLV121 virus (FDDV4) passaged in FDD cells and the 13th-generation DLV121 virus (FDDV13) passaged in FDD cells. In addition, the evolution of the LTR in viruses from horses infected with LN40, DLV121, or FDDV13 was analyzed by sequencing.

**FIG 2:**
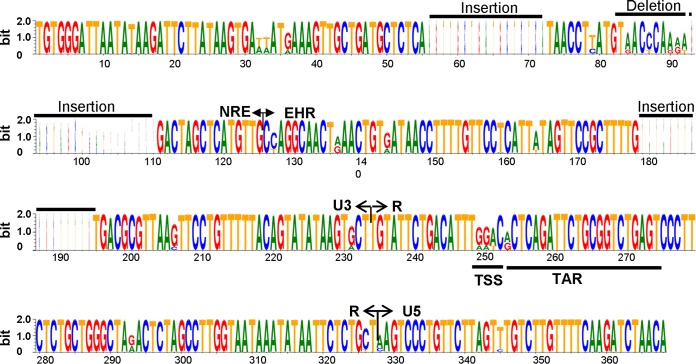
WebLogo presentation of the variability of the EIAV LTR. The height of each stack corresponds to the level of nucleotide conservation at that position. When the nucleotide is invariant, only one letter is shown; when the nucleotide is variable, the most common substitutions are noted. The figure depicts the main mutation hot spots in the LTR and the characteristics of base transformation. NRE, negative regulatory element; EHR, enhancer region; TSS, transcriptional start site; TAR, *trans*-activation responsive element.

To evaluate the diversity and evolutionary patterns of EIAV LTR quasispecies in different replication environments, this study followed the development of attenuated EIAV vaccines, including important strains (*in vitro*) during attenuation and their samples collected from infected horses (*in vivo*) (Fig. 1). A total of 149 *in vitro* clones and 795 *in vivo* clones covering virulent, adapted, and vaccine strain EIAVs were collected during attenuation and infection. The complete LTR sequences were amplified by PCR from each sample, a total of 13 to 25 clones were obtained from each sample, and a total of 944 sequences were aligned (see Fig. S1 to S4 in the supplemental material). The general variability characteristics of the EIAV LTR are highlighted in Fig. 2. We found 17 major point mutations in the LTR and 3 major insertion/deletion mutations in the U3 region, findings consistent with the results obtained from our previous investigation of *in vitro*-adapted EIAV strains (2, 16), but much more informative. These mutations resulted in changes in the U3 region binding sites of a number of transcription factors, including AP-1, methylation-dependent binding protein (MDBP), PU.1, and the basic helix-loop-helix (bHLH) transcription factor (to which the E-box binds) (Table 1).

**TABLE 1:** Characterization of transcription factor binding sites of various LTRs

Type of passage^*a*^	Sample (no. of clones detected)	No. of clones with the indicated transcription binding factor site^*b*^ in:										
		NRE region			EHR							
		AP-1	AP-1	AP-1	MDBP	E-box	PU.1	PU.1	PU.1	AP-1	PU.1	AP-1
*In vitro*	LN40 (20)	0	0	0	0	0	20	20	0	20	20	20
	DV117 (19)	0	0	0	1	0	19	19	0	19	19	19
	DLV34 (19)	0	0	0	5	16	19	19	0	18	19	19
	DLV62 (20)	4	0	0	8	14	20	20	0	20	20	20
	DLV92 (21)	17	0	0	8	16	21	20	0	21	21	20
	DLV121 (21)	21	0	2	7	17	21	21	10	21	21	20
	DLV137 (22)	13	0	16	3	18	21	21	4	22	22	22
	FDDV4 (23)	19	7	5	14	22	22	23	0	0	23	23
	FDDV13 (23)	4	23	23	18	23	23	23	0	23	23	23
*In vivo*												
LN40	25-14 (20)	2	0	1	1	2	20	18	0	20	20	20
	25-16 (19)	2	0	0	0	2	19	19	2	19	19	19
	25-28 (20)	0	0	0	0	0	20	20	0	20	19	20
	26-14 (13)	4	0	0	7	8	13	13	0	12	13	13
	26-18 (20)	0	0	0	0	0	20	19	0	20	20	20
	26-30 (18)	0	0	0	0	0	18	18	0	18	18	17
	4-14 (20)	5	0	0	3	6	20	20	5	20	19	20
	4-28 (20)	6	0	2	2	7	20	20	3	19	20	20
	4-42 (20)	3	0	7	2	5	20	20	0	20	20	19
	4-56 (20)	1	0	0	0	0	19	20	0	20	19	20
	4-70 (20)	0	0	0	0	0	20	20	0	20	20	20
	4-140 (22)	0	0	1	1	1	22	22	0	22	19	20
	10-28 (20)	5	0	6	9	12	20	20	0	20	19	20
	10-42 (19)	1	0	1	3	4	19	19	0	19	19	19
	10-56 (19)	0	0	0	0	0	18	19	0	19	18	19
	10-70 (21)	0	0	0	0	2	21	21	0	21	21	21
	10-84 (19)	0	0	0	0	0	19	19	0	18	19	19
DLV121	1-14 (19)	8	0	9	0	8	19	19	0	19	19	19
	1-28 (20)	8	0	8	0	8	20	20	0	20	16	20
	1-42 (20)	0	4	5	4	9	20	20	0	20	16	20
	1-56 (21)	0	0	0	0	0	21	21	0	21	21	21
	1-70 (20)	0	0	0	0	0	20	20	0	20	20	20
	1-84 (21)	5	0	0	3	3	21	21	0	21	21	21
	1-112 (15)	2	0	10	4	7	15	15	0	15	15	15
	1-140 (19)	9	0	0	3	13	19	19	0	19	19	19
	2-14 (21)	10	3	21	3	13	21	21	0	19	15	20
	2-28 (19)	3	0	0	3	2	18	18	0	18	18	17
	2-42 (19)	0	0	0	0	0	19	19	0	19	19	19
	2-56 (19)	0	0	0	0	0	19	19	0	19	19	19
	2-70 (21)	0	0	0	0	0	21	21	0	21	21	21
	2-84 (20)	1	0	0	0	1	20	20	0	20	20	20
	2-112 (22)	4	0	10	10	8	22	22	10	22	21	22
	2-140 (22)	5	0	7	2	7	22	22	3	22	22	22
FDDV13	6-15 (18)	2	16	16	18	18	18	18	0	18	18	18
	6-60 (13)	3	0	5	3	4	13	13	0	13	13	13
	6-120 (18)	0	12	16	11	12	18	18	4	18	18	18
	6-180 (20)	3	1	19	2	19	20	17	0	17	19	20
	8-15 (21)	0	0	21	0	19	21	21	0	5	20	21
	8-180 (18)	16	0	2	15	18	18	18	0	16	18	18

a For *in vivo* passages, the virus is given.

b Transcription factor binding sites are listed in order of their position in the LTR sequences.

**FIG 3:**
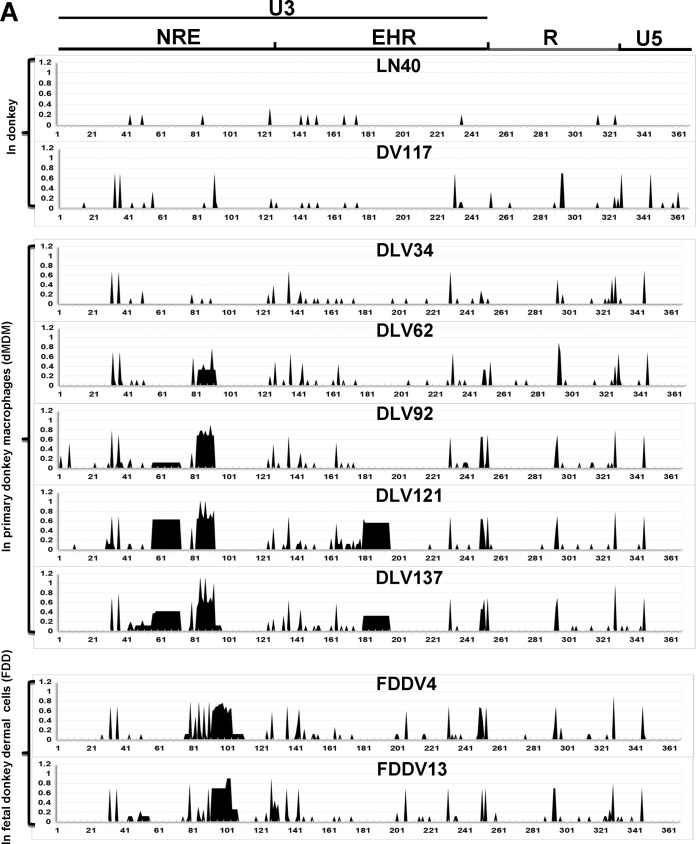

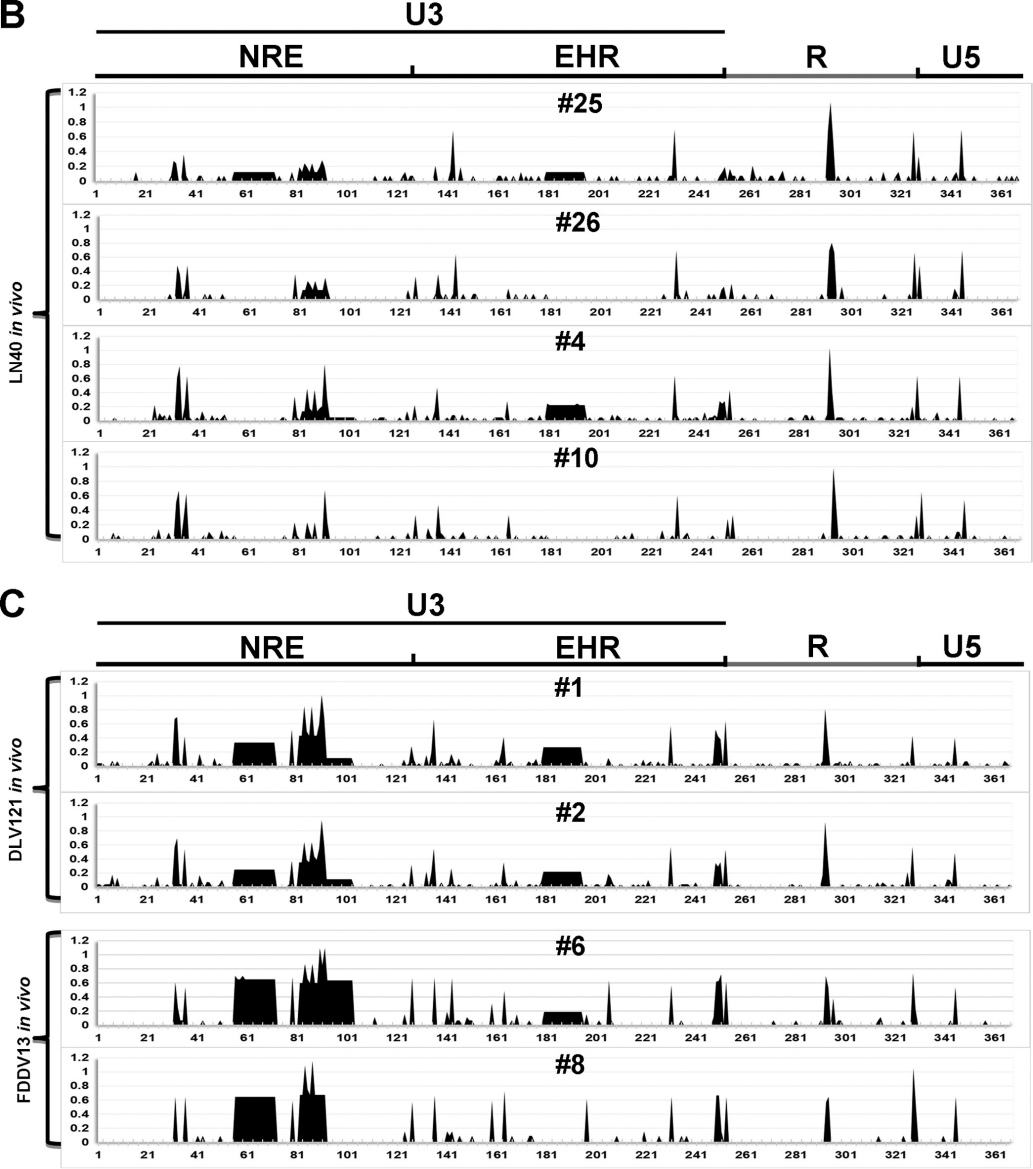
LTR variation patterns quantified by entropy values. For DV117, all samples from *in vitro*-adapted strains and from horses were compared with the full LN40 sequence, and entropy was calculated using BioEdit software. The entropy values are positively correlated with the mutation probability.

To intuitively characterize the variation in the LTR *in vitro* and *in vivo*, we used LN40 as a reference, and we compared and analyzed variation in various *in vitro* samples and in samples from horses infected with LN40, DLV121, or FDDV13 using the entropy value (Fig. 3). The entropy value reflects the frequency at which mutations occur in the LTR sequence. Overall, the EIAV LTRs from the *in vitro* and *in vivo* sources displayed the same major mutation sites (regions) and types of mutations but different mutation frequencies (Fig. 3; also Fig. S1 to S4 in the supplemental materials). Differences in the replication environment (*in vivo* or *in vitro*) or in the target cell species (dMDMs or FDD cells) resulted in increased or decreased numbers of transcription factor binding sites (Table 1). Notably, the same strain displays changes in binding site number for different transcription factors at different infection stages of the same individual or during the same infection stage of different individuals. The features of *in vitro* and *in vivo* evolution of the EIAV LTR are analyzed in detail below based on the patterns of the changes in the insertion/deletion mutations and transcription factor binding sites in the U3 region and in the TSS and TAR initiation sites in the R region.

### Characterization of EIAV LTR variability over the course of the development of an attenuated EIAV vaccine.

LN40 and DV117 were the virulent parental strains of the attenuated EIAV vaccine. These strains were isolated in the first febrile episodes of an experimentally infected horse and donkey, respectively. In reference to the LTR sequence of LN40, DV117 displayed more than 10 mutation sites, 7 of which had a high mutation frequency and were well preserved in various representative strains during *in vitro* passaging (dMDMs or FDD cells) (Fig. 3A; also dark shading in Fig. S1 in the supplemental material). These mutations were also present at high proportions in clonal isolates from horses subsequently infected with LN40 in this study (Fig. S2). Therefore, the DV117 and LN40 sequences used here are considered to represent only a quasispecies of the EIAV at a specific *in vivo* time point.

The dMDM-adapted strains originating from passage 62 (DLV62) and thereafter (DLV92, DLV121, and DLV137) had large insertion/deletion mutations in the U3 region, and the proportion of strains with these mutations gradually increased with the increase in the passage number (light shading in Fig. S1 in the supplemental material). The mutations included two insertion mutations in the NRE at positions 56 to 72 and in the EHR at positions 179 to 194 (containing a PU.1 binding site, which increased the number of EHR PU.1 binding sites to four) and deletion mutations or multiple point mutations at positions 82 to 92, which contain an AP-1 site. The two inserted fragments are both direct repeats of their 5′ adjacent sequences and might be involved in viral adaptation to cultured dMDMs.

The FDD cell-adapted virus FDDV4 lacked the insertion mutations characteristic of viruses passaged in MDMs, but the LTR sequences included insertions at positions 93 to 103 or 93 to 110 in the NRE region. This finding indicated that mutations in the U3 region were associated with adaptation to different target cell microenvironments. Multiple point mutations were found at positions 82 to 92, which were also identified in dMDM-adapted DLV121 and DLV137. The substitution mutations in FDDV4 formed one or two new AP-1 sites, either alone or combination with the 3′ inserted fragments. In FDDV13, most of the sites with multiple substitutions at positions 82 to 92 disappeared (including the AP-1 binding sites formed separately and by adjacent substitutions), whereas the insertions at positions 93 to 103 or 110 remained consistent and formed an AP-1 site in the initiation site (unshaded area in Fig. S1 in the supplemental material). These results suggest that the LTR should contain an AP-1 binding site in the NRE region to enable viral adaptation to FDD cells.

**FIG 4:**
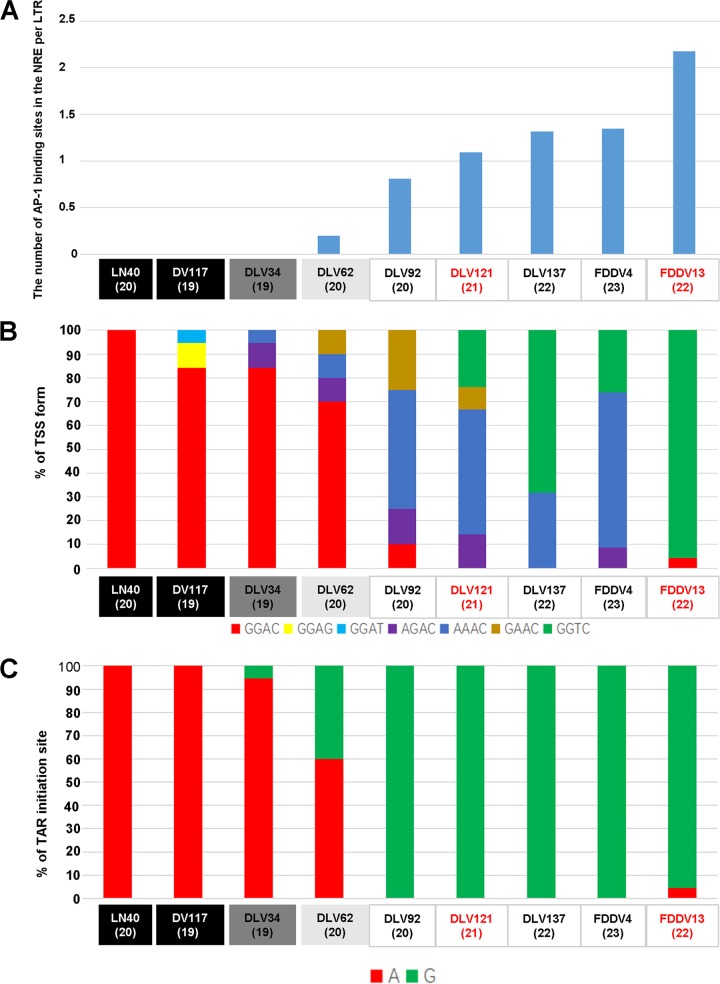
Mutations in the LTR resulted in a change in the quantity or type of functional sites. (A) The number of AP-1 binding sites in the NRE region per LTR gradually increased during the vaccine development process. (B) The ratios of the seven types of TSSs (color-coded as explained below the graph) were significantly changed at the different stages of vaccine development. (C) The predominant nucleotide of the TAR initiation site shifted from A to G with the attenuation of the host strain. Numbers of LTR clones are given in parentheses.

AP-1, which is a heterodimer of c-Fos and c-Jun, regulates cell differentiation, proliferation, and apoptosis (24). The NRE regions of LN40 and DV117 do not contain AP-1 binding sites. However, with the exception of DLV34, one to three AP-1 binding sites were commonly found in the NRE regions of all other *in vitro*-adapted strains. With virus passage *in vitro*, the proportion of strains with AP-1 binding sites in the NRE region gradually increased (Fig. 4A; Table 1; Fig. S1 in the supplemental material), suggesting that AP-1 binding sites were associated with reduced virulence.

In addition to increasing numbers of mutations in the U3 region, the frequency of mutations in the TSS and TAR initiation site of the R region gradually increased as the virulence of the strains decreased (Fig. 4B and C). The LTR TSS is present in seven main forms: GGAC, AGAC, AAAC, GAAC, GGAG, GGAT and GGTC. All of the LN40 clones had only GGAC TSSs. Although the TSSs of other virulent strains (DV117, DLV34, and DLV62) were still predominantly GGAC, the percentage of this form gradually decreased to <10% during *in vitro* passage. Moreover, the conversion from A to G in the TAR initiation site could be clearly observed during the evolution from virulent to vaccine strain EIAVs.

**FIG 5:**
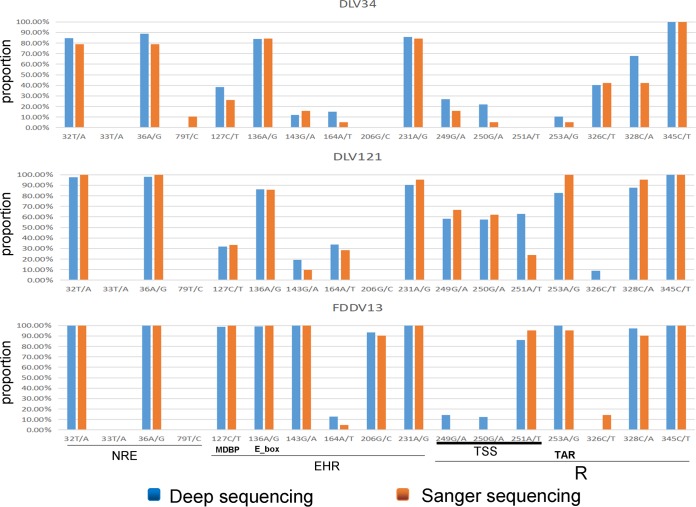
Comparison of the proportions of major variants in the LTRs as determined by deep sequencing and by Sanger sequencing. NRE, negative regulatory element; EHR, enhancer region; TSS, transcriptional start site; TAR, *trans*-activation responsive element; MDBP, methylation-dependent binding protein binding site; E_box, bHLH transcription factor binding site.

To confirm the frequency of occurrence of the major mutations described above among the different viral strains, as determined by Sanger sequencing, the proviral genomes of DLV34, DLV121, and FDDV13 were resequenced by deep sequencing. The frequencies of occurrence of the major variable sites within the LTRs of the three strains were compared with the frequencies obtained using deep-sequencing methods. Sanger sequencing and deep sequencing showed consistent results in the overall frequency trend of variable sites among the three viral strains (Fig. 5). Therefore, the Sanger method results objectively reflected the variation characteristics of EIAV.

### Evaluation of the promoter activity of the major LTR mutations in eMDMs and FDD cells.

**FIG 6:**
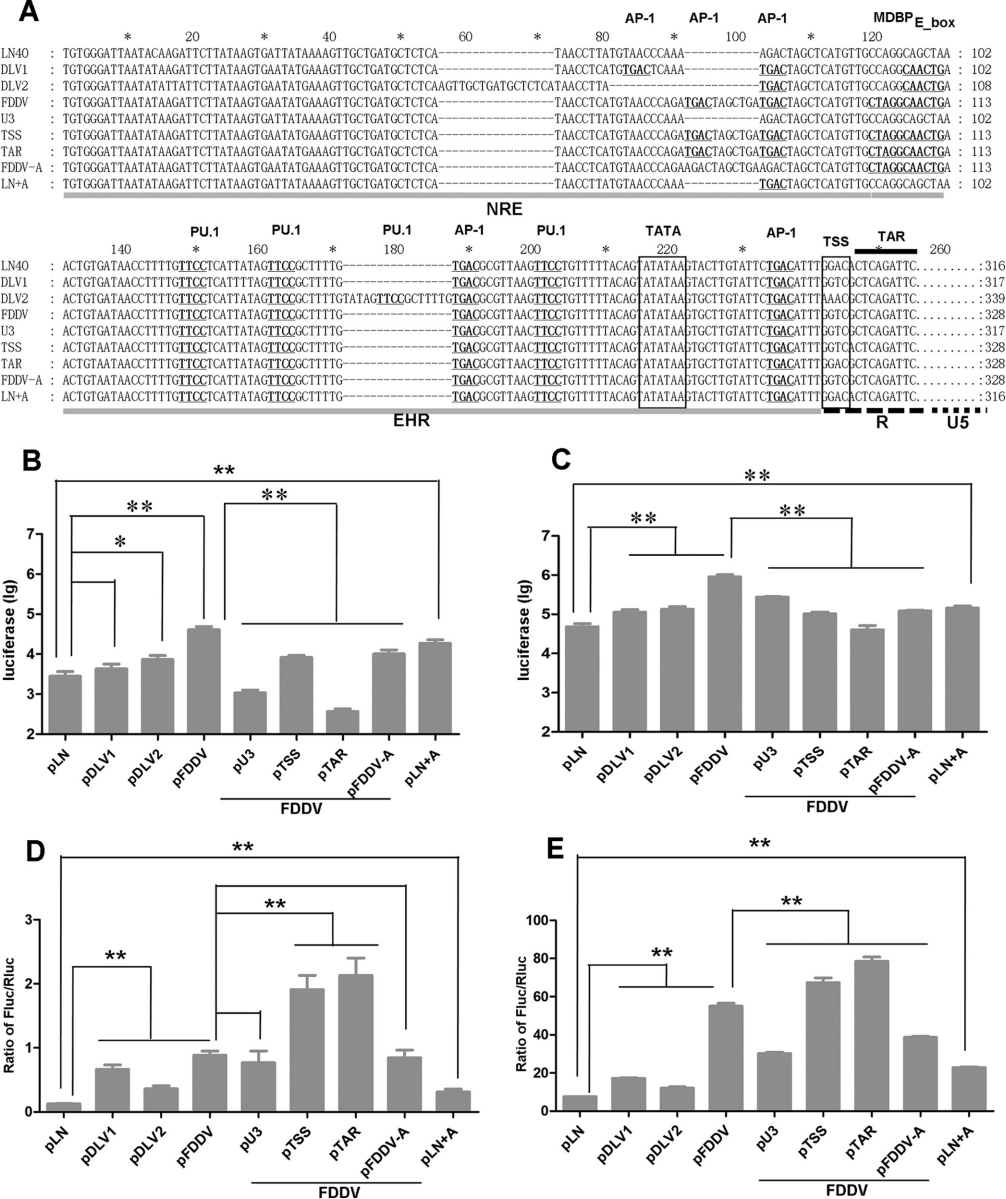
Effects of LTR mutations on the promoter activities of reporter genes *in vitro*. (A) Comparison of the different LTR variant sequences for which reporter luciferase gene plasmids were constructed. The luciferase reporter viruses were used to infect eMDMs without (B) or with (C) coinfection with DLV121 (for the provision of Tat). After 48 h, the cells were lysed, and the promoter activity of each LTR variant was examined. FDD cells were cotransfected with the pGL-LTRs, pRL-CMV vector, and pcDNA3.1 (D) or pTat (E). The cells were lysed 48 h posttransfection, and the firefly luciferase (Fluc) and Renilla luciferase (Rluc) activities were assayed. LN, derived from the LN40 LTR sequence, was used as the reference sequence. DLV1 was derived from DLV121; compared with the LN sequence, the NRE region of DLV1 has two additional AP-1 binding sites, the EHR has an additional E-box binding site, TSS is mutated to GGTC, and the TAR initiation site is mutated to G. DLV2 was derived from DLV121; compared with the LN sequence, its U3 region has two insertion mutations in positions 56 to 72 and 179 to 194 and a deletion mutation in positions 82 to 92, its NRE has an additional AP-1 binding site, the EHR has one additional E-box binding site, the TSS is mutated to GGTC, and the TAR initiation site is mutated to G. FDDV was derived from FDDV13; compared with the LN sequence, its NRE has an additional 11-bp repetitive sequence in positions 93 to 103 and two AP-1 binding sites, its EHR has one additional MDBP and one additional E-box binding site, the TSS is mutated to GGTC, and the TAR initiation site is mutated to G. U3 was based on FDDV, with the U3 region replaced with the LN sequence. TSS was based on FDDV, with the TSS mutated from GGTC to GGAC. TAR was based on FDDV, with the TAR start site mutated from G to A. FDDV-A was based on FDDV, with the 75T/A and 86T/A mutations and the absence of the two AP-1 binding sites in the NRE. LN+A was based on LN with the 75A/T mutation and the addition of one AP-1 binding site.

To examine the promoter activity of the LTR mutants, we selected nine representative hot variable regions for the construction of luciferase reporter plasmids. The characteristics of the nine sequences are illustrated in Fig. 6A. The basal promoter activity of pDLV1 (a DLV121 clone) was not significantly different from that of pLN (from LN40) in equine monocyte-derived macrophages (eMDMs), whereas the activities of pDLV2 (another clone from DLV121) and pFDDV (mimicking the FDD cell-adapted mutations) were 2.6 and 14.5 times higher than the activity of pLN in eMDMs (Fig. 6B). The promoter activities of pDLV1, pDLV2, and pFDDV were 2.4-, 2.8-, and 18.9-fold higher than the activity of pLN in eMDMs in the presence of Tat (Fig. 6C). Additionally, the activities of pDLV1, pDLV2, and pFDDV were 1.6- to 7.3-fold higher than the activity of pLN in FDD cells in both the absence and the presence of Tat (Fig. 6D and E). pFDDV displayed the highest promoter activity regardless of the presence or absence of Tat. When the U3, TSS, and TAR sequences of pFDDV were mutated to the pLN sequences (resulting in pU3, pTSS, and pTAR, respectively), the promoter activity of the LTR decreased significantly in eMDMs but increased in FDD cells, with the exception of pU3 (Fig. 6B to E).

To verify the effect of the presence of AP-1 binding sites in the NRE region on LTR promoter activity, we used mutagenesis by PCR to introduce one AP-1 binding site into the NRE region of pLN (resulting in pLN+A) and to eliminate two of the AP-1 binding sites from the NRE region of pFDDV (resulting in pFDDV-A). The results showed that the promoter activity of pLN+A increased significantly in eMDMs and FDD cells, whereas the promoter activity of pFDDV-A decreased significantly in FDD cells in the presence of Tat and in eMDMs (Fig. 6B to E).

These results indicate that mutation hot spots of the LTR benefit EIAV replication in permissive cells *in vitro* and that the promoter activity of the LTR is under comprehensive regulation by the U3 (including NRE and EHR), TSS, and TAR regions.

### Characterization of LN40 LTR variability *in vivo*.

Although the LTR of LN40 was highly conserved, significant sequence variations were observed after the virus infected and proliferated in horses (Fig. S2 in the supplemental material). Typically, the characteristics of the main variation sites were consistent with those obtained from the *in vitro*-adapted strains (Fig. 3A and B; also Fig. S2). Three noticeable evolutionary features of the virus were found. (i) During the early stage of LN40 infection (within 1 month), 10% to 45% of the clones had dMDM-adapted features (including insertion/deletion mutations in the U3 region and TSS and TAR initiation site mutations). In addition, during the early stages of inoculation, the samples obtained from horses 4 and 10, who were chronically infected with EIAV, had higher percentages of characteristic dMDM-adapted sequences (35% and 45%, respectively) than other samples. (ii) Most of the clones obtained at the typical acute clinical stage (samples 25-28 and 26-30) were identical to the LN40 sequences. (iii) Most clones isolated from horses chronically infected with EIAV (horses 4 and 10) were identical to the DV117 sequences. We did not observe typical EIA symptoms (fever, thrombocytopenia, and increased viral load) at the end of the experiment at day 140 (25).

### Characterization of EIAV vaccine LTR variability *in vivo*.

After inoculation of the horses, the frequency of dMDM-adapted mutations in the vaccine strain DLV121 was significantly reduced, and some clones began to show FDD cell-adapted features (Fig. 3C; also Fig. S3 in the supplemental material). Specifically, among the LTR clones from horses infected with DLV121 for 2 weeks (samples 1-14 and 2-14), 78% (15/19) and 100%, respectively, still retained some or all of the *in vitro*-adapted mutations; 19% (4/21) of the clones of sample 2-14 showed FDD cell-adapted features, and most of the clones displayed a certain frequency of reversions to the L40 or DV117 sequence. In the clones with reversion mutations, loss of the insert occurred significantly more frequently than reversion of the TSS or TAR initiation site. Surprisingly, most of the clones from samples obtained 4 to 12 weeks after infection were identical to the conserved sequences of DV117 or LN40. In contrast, 82% (18/22) of the clones from horse 2 at the 112-day sample time point had reverted to the DLV121 sequences, and 74% (14/19) of the clones obtained from horse 1 at 140 days postimmunization showed the characteristics of attenuated strains. Although approximately 90% of the clones obtained from horse 2 at 140 days postimmunization had reverted to conserved LN40 sequences, this horse did not display symptoms of EIA before or 2 months after challenge at 140 days postimmunization, whereas all three control horses not preimmunized with DLV121 developed acute EIA (25).

FDDV13 is an FDD cell-adapted strain that was derived by 13 passages of DLV121 in FDD cells. Interestingly, when measured 15 days after the initial infection of horses with FDDV13, the LTR sequences in some (14/18 clones in horse 6) or all (horse 8) clones had changed to dMDM-adapted features. No sequences with FDD cell-adapted features were detected in horse 8 at days 15 and 180 postinoculation. Sequences with FDD cell-adapted features were also not detected in the day 60 sample from horse 6 (sample 6-60), but 67% (12/18) and 5% (1/20) of the clones in the day 120 and day 180 samples (samples 6-120 and 6-180), respectively, from this animal had mutations specific for FDD cell-adapted viruses (Fig. S4 in the supplemental material). These findings suggest that quasispecies with LTRs adapted to dermal cells have difficulty adapting to the *in vivo* environment, although a very low proportion of these viruses can still enter replication or latency in the *in vivo* environment.

### Changes in the LTR variation of EIAV quasispecies *in vitro* and *in vivo*.

**FIG 7:**
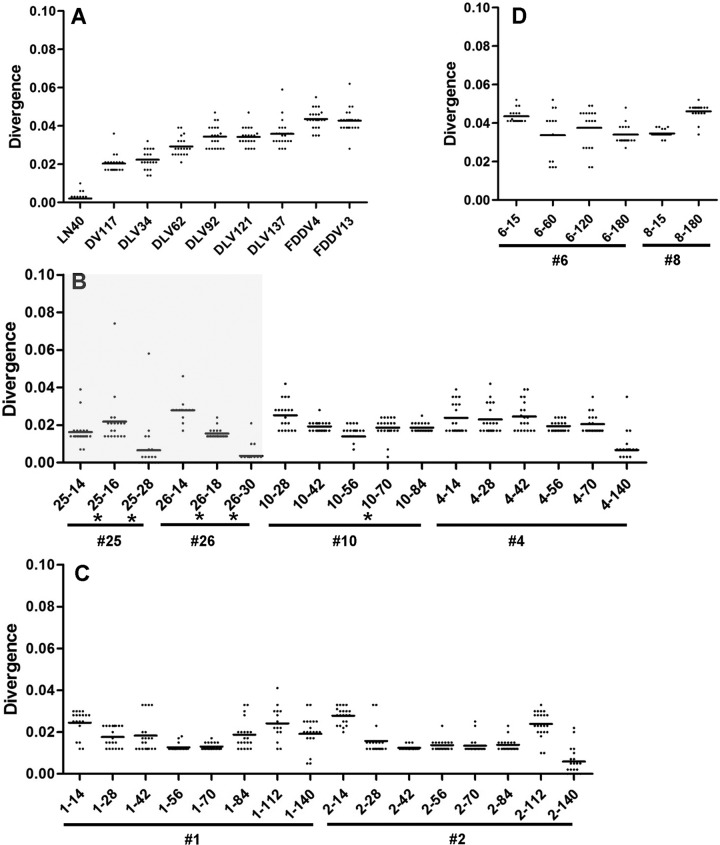
Analysis of the LTR quasispecies after long-term replication in different environments. Divergences among LTR sequences were calculated for strains that were successively passaged *in vivo* and *in vitro* as presented in Fig. 1 (A) and for strains isolated at different stages from horses inoculated with the virulent LN40 strain (B) or the vaccine strain DLV121 (C) or FDDV13 (D). The divergence plot shows changes in the LTR quasispecies populations from the consensus LN40 sequence prior to inoculation. Asterisks indicate horses with fever (39°C). Identification numbers corresponding to individual horses are preceded by number signs (#). The shaded area indicates samples from horses with typical disease.

The analysis presented above demonstrated that the LTR displayed a high degree of heterogeneity. To better reflect the variability of the EIAV LTR in quasispecies, we analyzed the divergence of all LTR sequences using the LN40 consensus sequence as the reference, as shown in the histograms in Fig. 7. Generally, the divergence was increased by *in vitro* passage (Fig. 7A). Interestingly, in clones isolated from horses 25 and 26, which were infected with LN40 and developed typical disease, a sharp decrease in divergence from the asymptomatic stages to the febrile stage was observed, indicating a potential correlation between the dominant quasispecies conversion and the host disease (Fig. 7B, shaded area); in clones isolated from horses 4 and 10, which had subclinical infections without any clinical symptoms, relatively stable divergence was observed, indicating a gradual evolutionary process of the EIAV quasispecies under a subclinical status (Fig. 7B, unshaded area). A significant fluctuation in divergence can be observed in the clones isolated from the DLV121- and FDDV13-inoculated horses, which may suggest the presence of a convertible dominant quasispecies (Fig. 7C and D).

### Phylogenetic analysis of the EIAV LTR.

**FIG 8:**
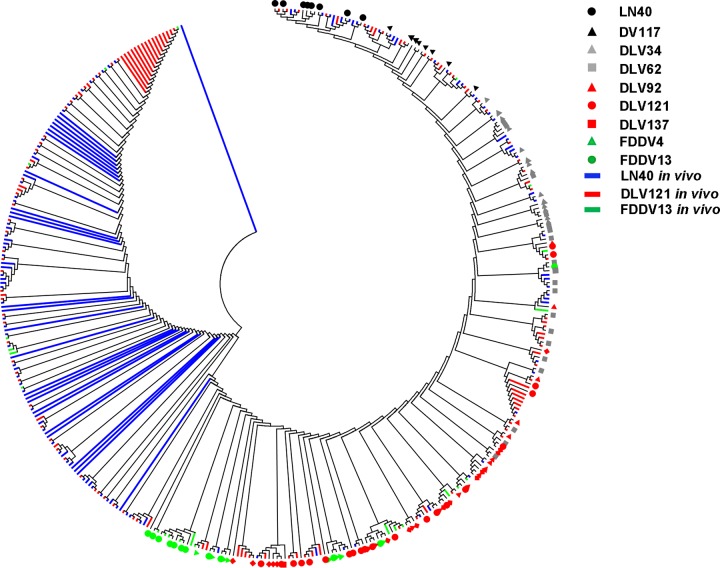
Phylogenetic analysis of the LTR sequences of EIAV strains replicated either *in vitro* (represented by symbols with different shapes and colors) or *in vivo* (represented by lines with different colors). The phylogenetic tree was constructed by the neighbor-joining method calculated with the Kimura 2 parameter in MEGA 6.0 software.

To systematically analyze the evolution of the LTR *in vitro* and *in vivo*, we used the CleanCollapse analytical program to merge the identical sequences of each sample into a single sequence and then clustered all 596 complete LTR sequences into a phylogenetic tree. As shown in Fig. 8, the sequences of LN40, DV117, and the *in vitro*-passaged strains were mostly distributed in the right half of the phylogenetic tree and were roughly arranged in the order of virulence attenuation shown in Fig. 1. However, the sequences of the *in vitro*-passaged strains did not form an independent branch. There was significant overlap between adjacent generations and even between nonadjacent generations, displaying a pattern of gradual transition. This phenomenon suggests that *in vitro* evolution of the virus involves the gradual accumulation of dominant quasispecies. In addition, the distribution of sequences from horses infected with LN40, DLV121, or FDDV13 was found in almost all branches of the phylogenetic tree, indicating a high degree of LTR variation *in vivo*. In particular, a clustering branch involving both clones of *in vitro*-adapted EIAVs and clones isolated from LN40-infected horses was found, indicating that the *in vitro*-adapted EIAV might be preexisting in the natural EIAV quasispecies.

## DISCUSSION

RNA viruses exist as complex quasispecies that contain multiple variants, and the proportions or amounts of each variant differ with the host microenvironment (22). This study followed the process of development of the attenuated EIAV vaccine by systematically analyzing the features of LTR evolution during the passage and attenuation of the virulent strain (LN40) *in vitro* and *in vivo*. Here, we carried out a study with two dimensions covering 149 *in vitro* clones and 795 *in vivo* clones, including virulent, adapted, and vaccine EIAVs, using time-dependent follow-up. One dimension followed the attenuation of virulence from virulent EIAVs by passage to transitional adapted EIAVs and then to the final vaccine EIAVs, whereas the other dimension followed the conversion between *in vivo* and *in vitro* adaptation.

Previous studies using American EIAV stains showed a different pattern of LTR evolution. Maury et al. showed high variation in LTRs from different time points of an EIAV-infected individual (17). Studies from the Montelaro laboratory showed highly conserved LTR sequences of EIAV isolated from different organs of horses with infections spanning one and a half years (18). In a study of the determination of virulence, Payne et al. found that LTR mutation contributed to viral replication and virulence (3, 19). In this study, we found highly variable sequences and multiple directions of LTR evolution *in vitro* and *in vivo* by analyzing a large number of LTR sequences. Although there was a great disparity between the sequences of Chinese EIAV strains and American strains or other strains (15, 26), with different types of transcription factor binding sites, in general the virus replicated in the same pattern, and our results provide a more comprehensive understanding of LTR evolution.

We found that the long-term *in vitro*-adapted viruses (most of which were at passage 62 or higher) often displayed multiple and distinct mutations at the EHR, NRE region, TSS, and TAR initiation site in the LTR that were parallel to the passage-dependent attenuation of the EIAVs; changes in these regions of the LTR could significantly affect its promoter activity (Fig. 6). These results are consistent with observations from our lab (27) and other labs (28, 29). Interestingly, *in vitro*-adapted mutations were found in the LN40-infected horses. In addition, a few weeks postinoculation, the *in vitro*-adapted forms of the LTR in the DLV121-immunized horses could be detected only at low levels, whereas the LTR clones characterized for the parental DV117 or LN40 strain became abundant. Moreover, FDDV13-immunized horses showed a significant shift toward MDM-adapted features. Furthermore, although FDD cells are not natural target cells for EIAV, FDD cell-adapted mutations were found in the dMDM-adapted strain DLV137, in LN40-infected horses, and in DV121-immunized horses (Fig. S1 to S3 in the supplemental material), indicating that FDDV-like clones were present in the viral pool due to the existence of FDD cell-like host cells. These results demonstrate that the primary source of dominant quasispecies in vaccines may not be clones arising from individual mutations but may instead be derived from an existing pool of quasispecies varieties. This hypothesis is supported by the phylogenetic analysis of all 596 unique LTR clones isolated *in vitro* and *in vivo* that is presented in this article, as well as by previous reports of *in vivo* evolution of the *gp90* and *S2* genes of LN40, which found that some viral clones isolated from certain phases of LN40-infected horses were identical or similar to the vaccine strains DLV121 and FDDV13 (30, 31). These strains may account for a small proportion of the LN40 viral quasispecies and may be selected in response to changes in the intracellular environment. Similarly, a portion of pathogenic quasispecies can remain in the attenuated vaccine strains. We showed previously that approximately 10% of *gp90* clones from the FDD cell-adapted strain FDDV13 were characteristic of LN40 (32). This result, indicating a complicated evolution of LTR *in vitro* and *in vivo*, and the appearance of these LTRs might not fully explain the virulence of viruses.

The dominant clones with dMDM-adapted LTRs reappeared at days 112 and 140 after clones were largely replaced with LN40-like sequences in the horses 4 weeks after immunization with DLV121 (Fig. S2 in the supplemental material). Furthermore, the LTR type became dMDM adapted in clones isolated from FDDV13-immunized horses. These observations indicate that EIAV strains with dMDM-adapted LTRs can establish persistent infections and become predominant *in vivo*, suggesting that cells similar or identical to MDMs exist *in vivo*. Moreover, the eventual transformation of the LTR from FDD cell adapted to dMDM adapted in FDDV13-inoculated horses could be explained if the cells supporting the replication of FDD-tropic viruses were poor in either numbers or permissiveness. HIV-1 acts predominantly as an R5-tropic virus during the early stage of infection of macrophages and dendritic cells. During disease progression, the virus transforms via changes in the gp120 envelope protein into a virulent X4-tropic virus that is highly infectious to naïve and memory CD4^+^ T cells (33), with the presence of a transitional R5/X4 dualtropic virus (34). Transformations in the tropism and virulence of HIV-1 are caused by changes in the proliferative activity of the target cells, changes in the subsets of cells that undergo immune activation after HIV-1 infection, and other factors, such as the distinct cellular niches presented by different target cells (35). The LTRs of EIAV are a major determinant of cell tropism (29), and although LTR variation may increase during the acute phase of EIA infection, studies have not reported a change in the cellular tropism of EIAV in the host (1, 3, 4, 10, 17, 18). The different *in vivo* evolutionary patterns of the LTR sequences of MDM- and FDD cell-adapted vaccine strains suggest that these two types of *in vivo*-adapted viruses may have different primary target cells after host infection and that these target cells may differ as a function of the infection time. Interestingly, all samples from the four LN40-infected horses included dMDM-adapted LTRs, especially during the early stages of infection, implying that changes in the target cells of EIAV might be a natural event during EIAV infection. Our data presented in this article and previous publications revealed that the dMDM- and FDD cell-adapted mutations were associated with the attenuation of EIAV virulence. However, whether the decreased pathogenicity was generated by a reduction of the viral capacity to replicate in the principal natural target cells or by the transformation of tropism for the target cells is not clear. In addition, we found that the proportions of MDM-adapted LTRs in viruses isolated during the early phase from horses with chronic EIA infections (horses 4 and 10; 35% and 45% of the viruses, respectively) were significantly higher than the proportions in viruses isolated from acute-onset horses (horses 25 and 26; 10% to 23% of the viruses, respectively). This difference in LTR type is promising, although whether this difference contributes to the direction of disease development is unclear.

**FIG 9:**
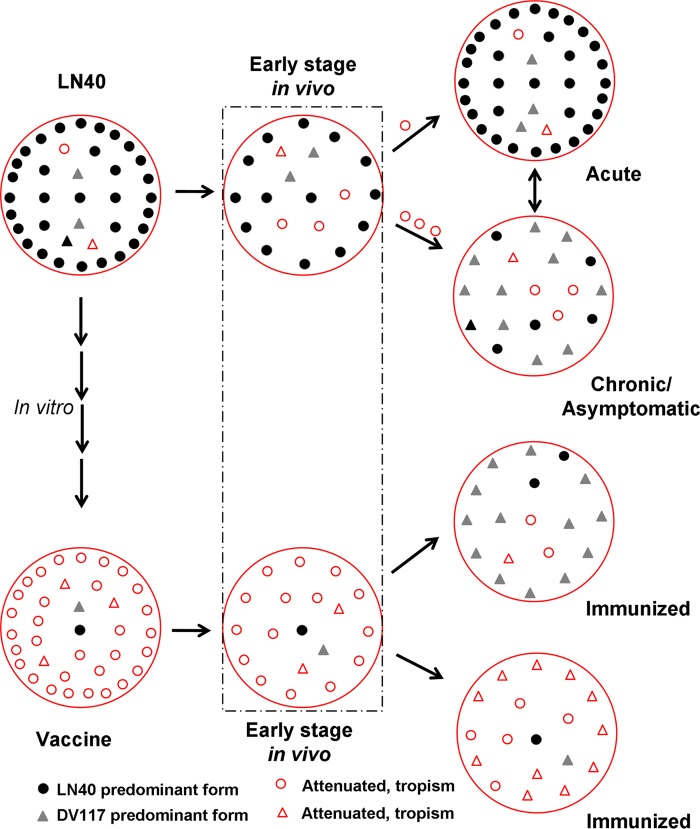
The quasispecies composition may affect viral pathogenicity. Large circles represent viral quasispecies with different population structures. The various symbols portray different viral particles as shown in the key. The population density represents the virus concentration.

In summary, this study characterized EIAV LTR evolutionary changes during the attenuation process by successive passaging in cultivated permissive cells and during the infection course after reentry into the host. These genomic shifts were generated in EIAV as a population of quasispecies with distinct diversity and divergence and were closely associated with viral pathogenicity. In particular, our results strongly suggest that the selection of the dominant EIAV quasispecies during adaptation to altered microenvironments is driven largely by the selection of viral clones that preexist as a very small population. Because DLV121-like clones were identified in the LN40 genomic reservoir, LN40-like LTR sequences were also detected from DLV121-inoculated horses. We presume that, as illustrated in Fig. 9, during infection with pathogenic EIAV strains, such as LN40, the dominant highly pathogenic quasispecies, which contains the LN40- or DV117-specific LTR, quickly overwhelms the other quasispecies, resulting in acute EIA. In some cases, the population of less-pathogenic quasispecies, which contains dMDM- or FDD cell-specific mutations, prevails, resulting in chronic or asymptomatic EIA. These situations are perhaps determined by either the amount and quasispecies composition of the EIAV inoculum (in agreement with the observations for horses 4 and 10) or the health and immune conditions of the host. The LTRs of the dominant quasispecies in the attenuated vaccine strains DLV121 and FDDV13 do not direct effective EIAV replication *in vivo*, and they generate quasispecies with low viral loads and wide diversity, especially in the earlier phases. Consequently, immunity was developed under the stimulation of the low-level replicated vaccine strains and subsequently controlled the replication of the pathogenic quasispecies, which may be temporally selected during long-term replication in the host. This immunity to EIAV matures together with the *in vivo* evolution of the inoculated virus and is able to prevent subsequent infection.

## MATERIALS AND METHODS

### Experimental infection and sample collection.

The Harbin Veterinary Research Institute, Chinese Academy of Agricultural Sciences, preserved all of the viral strains used in this study. The virulent strains LN40 and DV117 were isolated from the peripheral blood mononuclear cells (PBMC) of infected horses or donkeys during the febrile phase after infection with these viruses. Five representative viruses were sampled during the successive passages of DV117 in cultivated dMDMs and are named according to the generation of the passages (i.e., DLV34, DLV62, DLV92, DLV121, and DLV137). In addition, two representative viruses (FDDV4 and FDDV13) were sampled during the numbered passages of DLV121 in cultivated FDD cells. All EIAV blood samples were prepared from stocks from previous studies, including four horses infected with LN40 (horses 4, 10, 25, and 26) (25, 31), two horses infected with DLV121 (horses 1 and 2) (25), and two horses infected with FDDV13 (horses 6 and 8) (36). The rectal temperatures of the horses that received EIAV inoculation and their plasma EIAV viral loads and platelet counts were measured during the early stage of EIA clinical onset.

### DNA extraction, PCR and cloning.

EIAV LTR sequences were amplified and cloned from proviral DNA integrated into the host genomes. Genomic DNA was extracted, using the Omega blood DNA kit (Omega, USA), from peripheral blood cells of EIAV-infected horses or from dMDMs and FDD cells infected with various viral strains. The complete LTR sequence was amplified using primers LTR-L (TGT GGG AAT ATA AGA TTC T) and LTR-R (TGT TAG ATC TTG AAA ACA AGA C) with the genomic DNA from each sample as the template. PCRs with 30 cycles of amplification were performed for most samples, with the exception of those from the vaccinated horses (DLV121 and FDDV13) and asymptomatic LN40-infected horses. Due to the low viral load, PCR amplification of these samples was performed twice with 35 cycles per PCR. All samples were subjected to three to five independent PCRs. After purification by agarose gel electrophoresis, the PCR products were ligated to the pMD18-T vector (TaKaRa, Dalian, China), and 13 to 25 clones from each sample were randomly selected for sequencing.

The DLV34, DLV121, and FDDV13 proviral genomes were amplified in four fragments using PCR. The PCR was performed using PrimeSTAR HS DNA polymerase (TaKaRa, Dalian, China). All amplifications were performed in triplicate to obtain independent PCR products. The DLV34, DLV121, and FDDV13 proviral genomes were sequenced by deep sequencing using the Illumina HiSeq 2500 system (Axeq Technologies, Rockville, MD, USA).

### Sequence analysis.

All LTR sequences determined were first organized using the EditSeq tool in Lasergene DNAStar 7.1 software. The nucleotide sequence alignment was performed using ClustalW. To demonstrate the variability of the LTR as a whole, the online software WebLogo (http://weblogo.berkeley.edu/logo.cgi) was used to construct WebLogo diagrams. Entropy values were calculated and analyzed using BioEdit software. The molecular phylogenetic tree and genetic distance were constructed and analyzed using the neighbor-joining method with MEGA 6.0 software (37).

### Construction of EIAV LTR-driven luciferase gene expression vectors and analysis of promoter activity.

To analyze the promoter activity of the LTR variants in both FDD cells and MDMs, we constructed two sets of EIAV LTR-Luc plasmid systems. A set of luciferase reporter plasmids under the direction of different LTRs was constructed by cloning various LTRs into the pLG3-Basic vector (Promega) at the KpnI and XhoI sites (resulting in pGL-LTR constructs). The pGL-LTR constructs were transfected into FDD cells in 24-well culture dishes using the FuGene HD transfection reagent (Promega) and the following amounts of plasmids per well: 1 μg of the pGL-LTR plasmid, 0.02 μg of the pRL-CMV vector plasmid (Promega), and 0.5 μg of the pcDNATat plasmid (a plasmid expressing the Tat protein of FDDV13) or pcDNA3.1(+). The cells were lysed 48 h posttransfection, and the luciferase activity of the cell lysates was analyzed according to the instructions provided with the Dual-Luciferase reporter assay system (Promega, USA).

Because it is difficult to transfect microphages such as dMDMs with plasmid DNA, a three-plasmid lentiviral transfection system was constructed and used. The cytomegalovirus (CMV) promoter of the transfer vector pTY-CMV-luc was replaced by various LTR sequences (resulting in pTY-LTR-luc). The luciferase reporter virus was rescued using the method reported in the literature (38). Luciferase reporter viruses were rescued by cotransfection into 293T cells with pTY-LTR-luc; the transfer vector psPAX2 was included as a helper plasmid, and pMD2.0G was included to supply the coat protein VSV-G (vesicular stomatitis virus glycoprotein G). After 48 h, the culture supernatants were collected, centrifuged at 1,000 rpm for 10 min to remove cell debris, filtered through a 0.45-μm filter unit, and stored at −80°C. Each luciferase reporter virus was quantified using the HIV-1 p24 antigen ELISA kit (ZeptoMetrix Corporation, USA). eMDMs were seeded into 96-well plates at a density of 1 × 10^5^ cells per well. After 24 h, the same amount of each luciferase reporter virus was inoculated into two duplicate wells. To evaluate the promoter activity of the LTR in the presence of Tat, DLV121 (multiplicity of infection [MOI] = 5) was added 1 h prior to infection with the luciferase reporter viruses to provide the Tat protein. At 48 h after luciferase reporter virus infection, the supernatant was discarded; then the cells were lysed in 50 μl of lysis buffer and were assayed for luciferase activity according to the instructions provided with the luciferase assay system (Promega, USA).

### Statistical analysis.

Statistical analysis was conducted using GraphPad Prism, version 5 (Graph Pad Software, USA), and Excel 2016. Student's *t* test (two-tailed, with 95% confidence intervals) was used to compare differences between groups. Probability values less than 0.05 were considered statistically significant.

### Accession number(s).

The GenBank accession numbers determined in this study are as follows: for LN40 passaged *in vivo*, KY411969 to KY412098; for DLV121 passaged *in vivo*, KY465001 to KY465316; for FDDV13 passaged *in vivo*, KY465317 to KY465425; and for *in vitro*-adapted strains, HM141909 to HM141912, HM141919, HM141913 to HM141918, HM141920 to HM141923, AF327878, and GU385353 to GU385362.

## Supplementary Material

Supplemental material
