# Supplementary material for: Characterization of Equine Infectious Anemia Virus Long Terminal Repeat Quasispecies In Vitro and In Vivo
Source: J Virol. 2018 Mar 28;92(8):e02150-17. doi: 10.1128/JVI.02150-17 (PMC5874411; doi:10.1128/JVI.02150-17)
Supplement: Supplemental material [file supp_92_8_e02150-17__index.html]

Characterization of Equine Infectious Anemia Virus Long Terminal Repeat Quasispecies In Vitro and In Vivo — Supplemental material 

# Characterization of Equine Infectious Anemia Virus Long Terminal Repeat Quasispecies *In Vitro* and *In Vivo*

## Supplemental material

- Supplemental file 1 -

  Fig. S1 (Comparison of LTR sequences of various EIAV strains during the attenuation process.)

  Fig. S2 (Sequence comparison of LTRs from LN40-infected horses.)

  Fig. S3 (Sequence comparison of LTRs from DLV121-infected horses.)

  Fig. S4 (Sequence comparison of LTRs from FDDV13-infected horses.)

  PDF, 198K
