# Supplementary material for: Characterization of Equine Infectious Anemia Virus Long Terminal Repeat Quasispecies In Vitro and In Vivo
Source: J Virol. 2018 Mar 28;92(8):e02150-17. doi: 10.1128/JVI.02150-17 (PMC5874411; doi:10.1128/JVI.02150-17)
Supplement: Supplemental material [file JVI.02150-17_zjv008183460s1.pdf]

FIG S1， to be continued-1

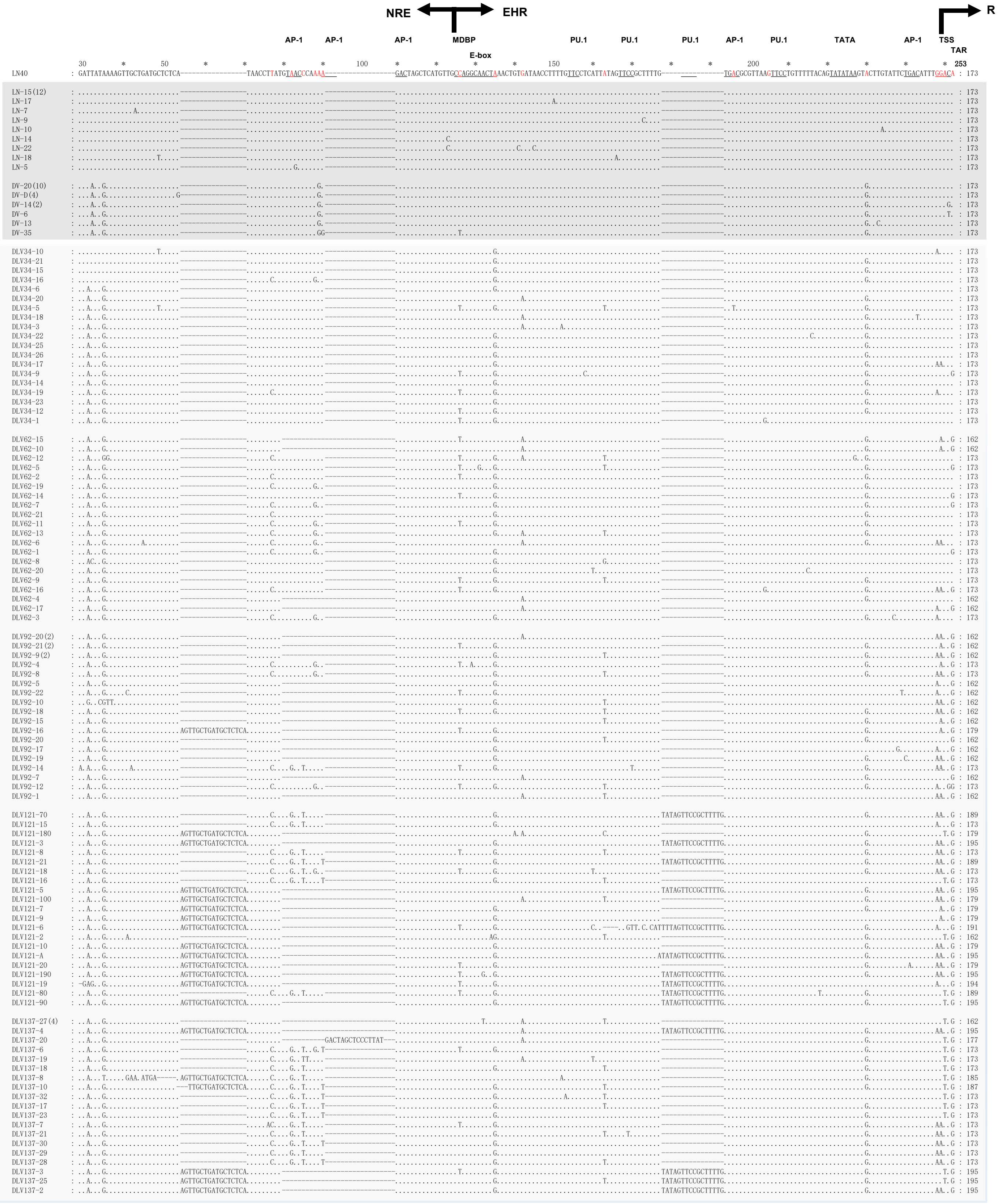

FIG S1, to be continued-2

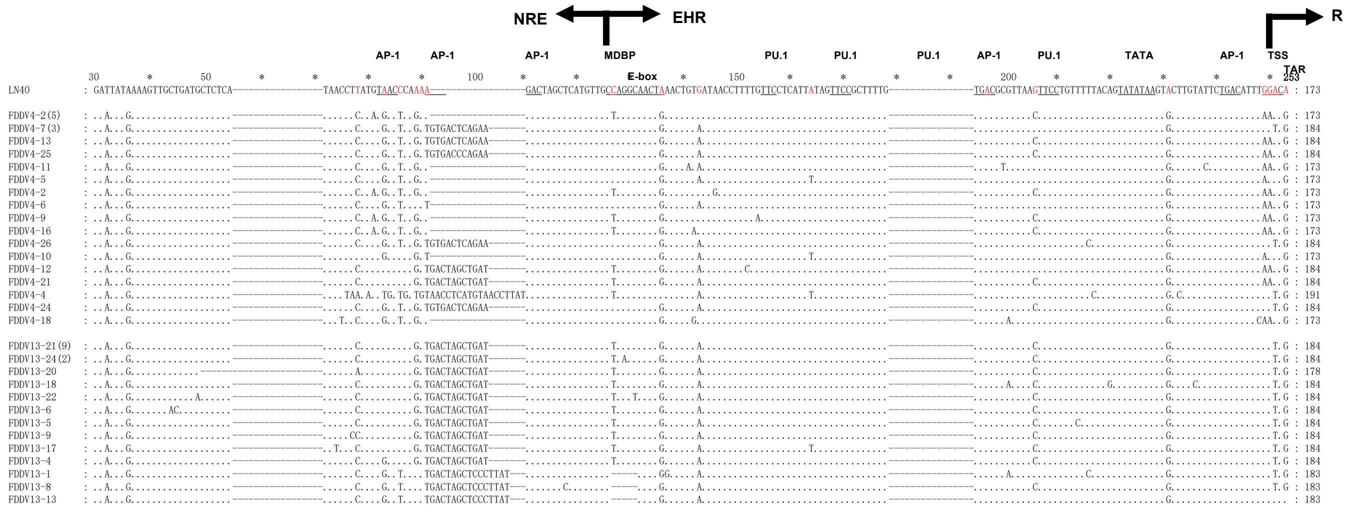

FIG. S1. Comparison of the LTR sequence of various EIAV strains during the attenuation process. All LTR sequences of the samples were aligned with the LN40 consensus sequence, which is the reference sequence. Nucleotides that are the same as those in the reference sequence are represented by dots, and nucleotides differing from those in the reference sequence are represented by the corresponding letter symbols; the dashes indicate deletion mutations. The numbers in brackets on the left side of the figure indicate the number of identical clones in each sample; the red letters in the top LN40 consensus sequence represent the main mutation hotspots of the LTR, and the underlined regions indicate the locations of the various transcription factor binding sites and the TSS.

FIG S2, to be continued-1

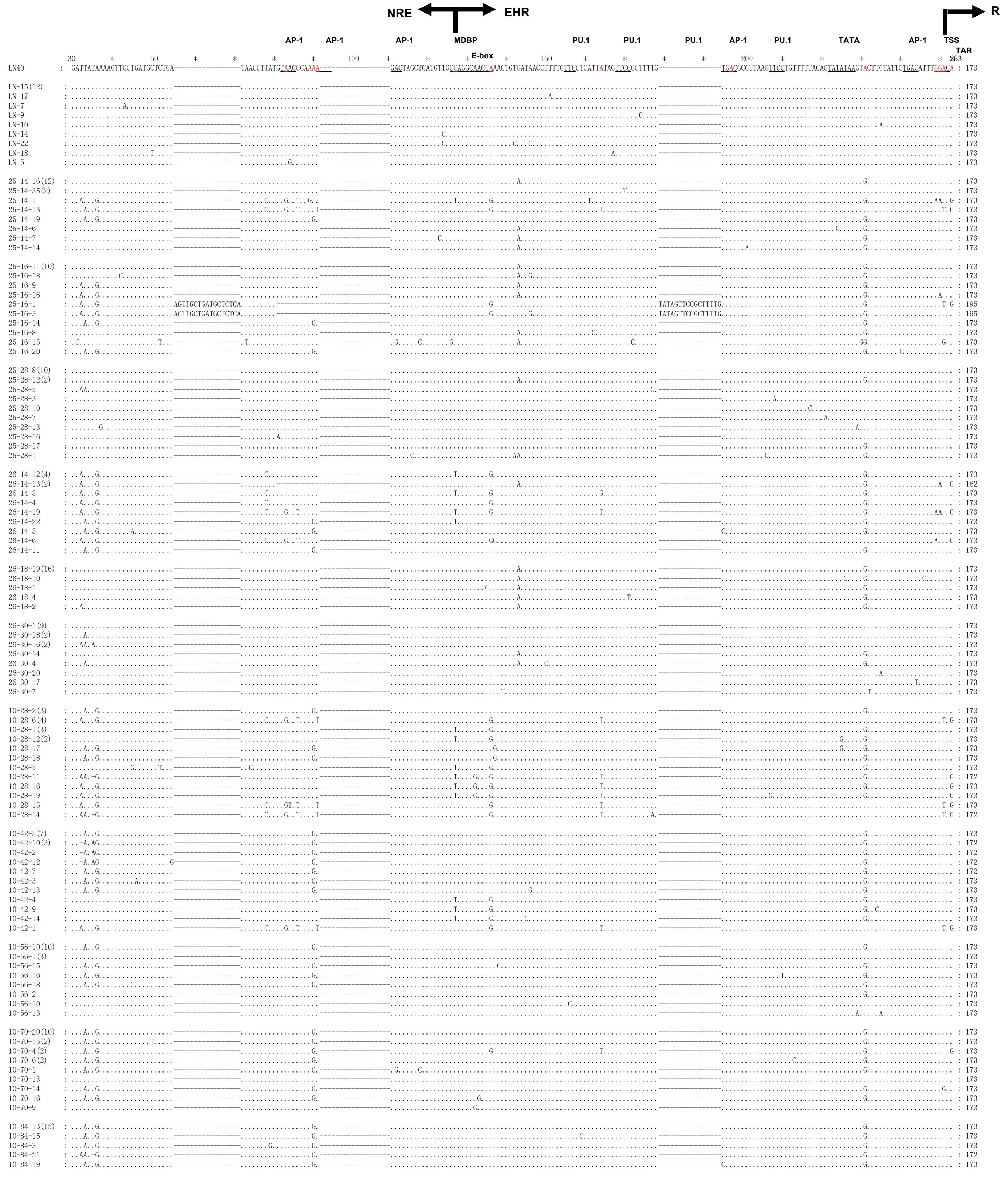

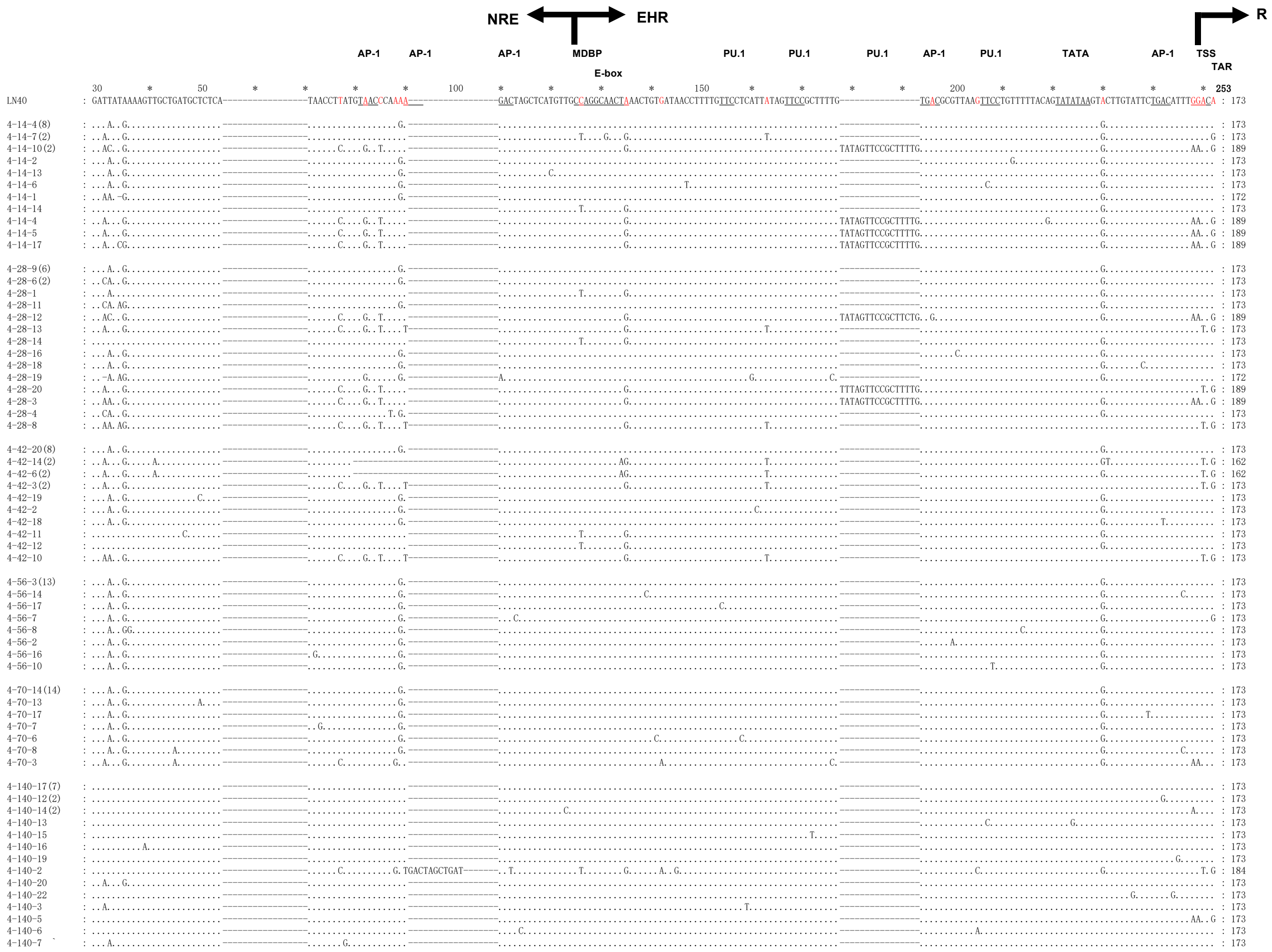

FIG. S2. Sequence comparison of LTRs from LN40-infected horses. All LTR sequences of the samples were aligned with the LN40 consensus sequence, which is the reference sequence. Nucleotides that are the same as those in the reference sequence are represented by dots, and nucleotides differing from those in the reference sequence are represented by the corresponding letter symbols; the dashes indicate deletion mutations. The numbers in brackets on the left side of the figure indicate the number of identical clones in each sample; the red letters in the top LN40 consensus sequence represent the main mutation hotspots of the LTR, and the underlined regions indicate the locations of the various transcription factor binding sites and the TSS.

FIG S3, to be continued-1

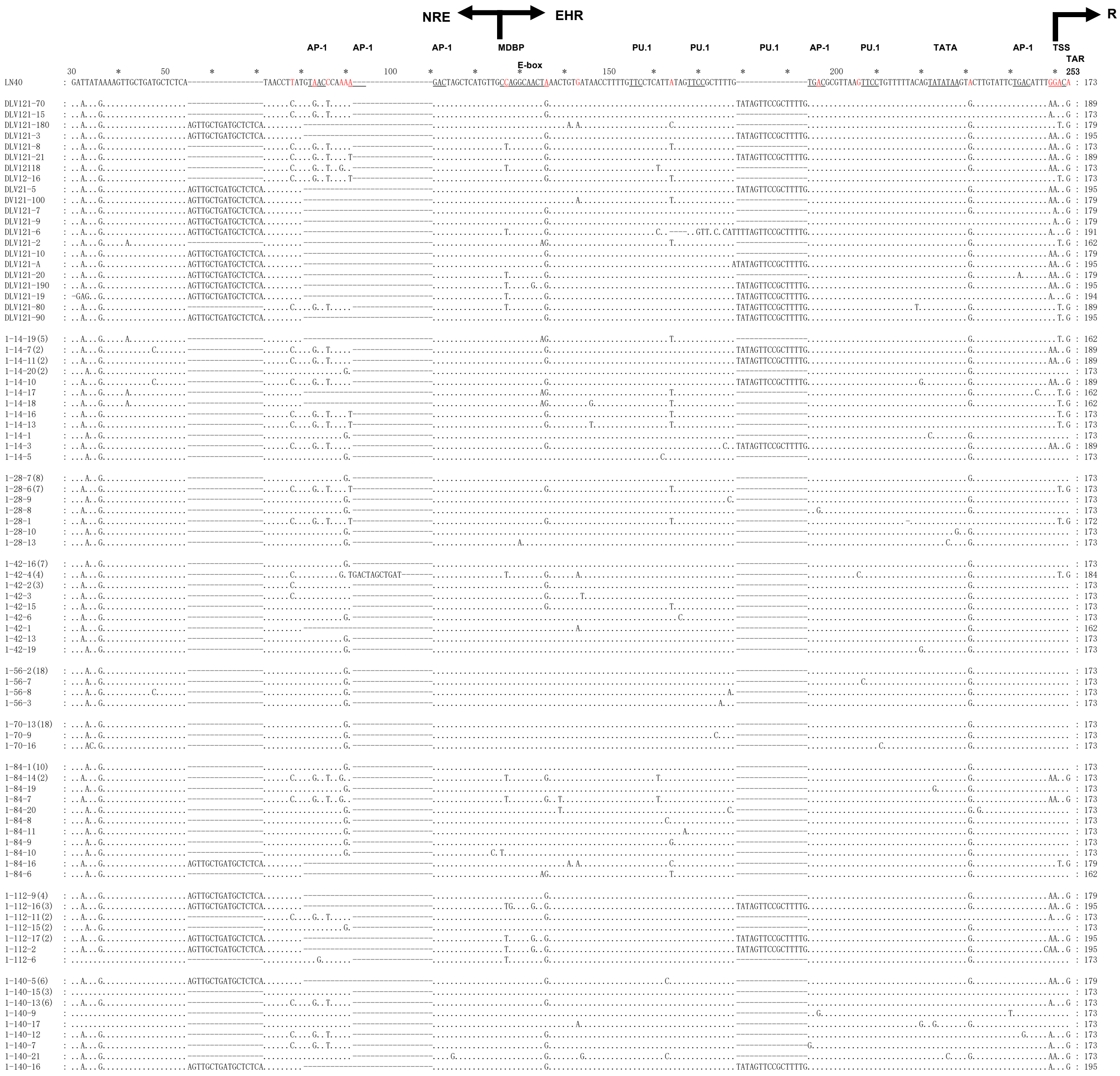

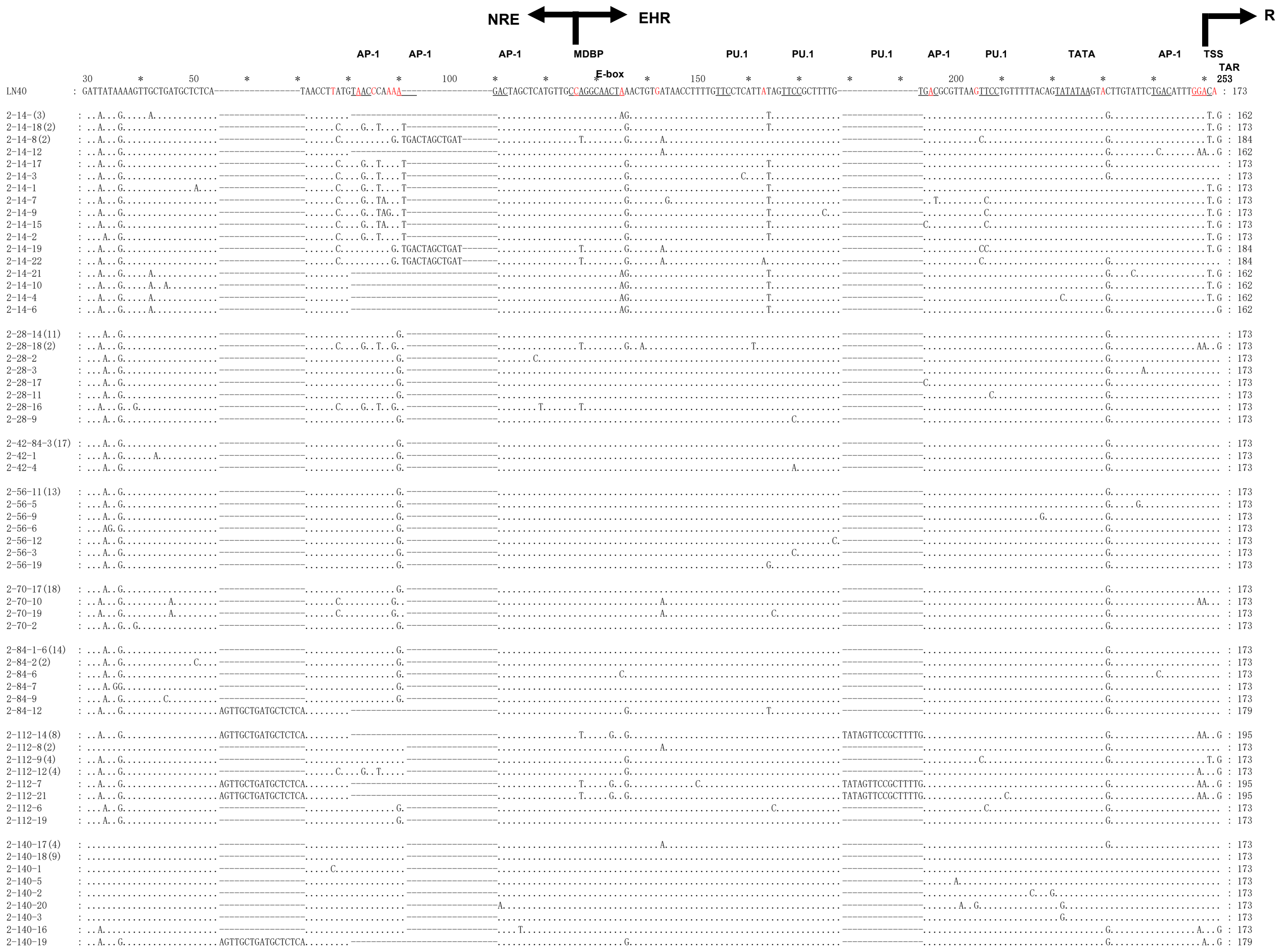

FIG. S3. Sequence comparison of LTRs from DLV121-infected horses. All LTR sequences of the samples were aligned with the LN40 consensus sequence, which is the reference sequence. Nucleotides that are the same as those in the reference sequence are represented by dots, and nucleotides differing from those in the reference sequence are represented by the corresponding letter symbols; the dashes indicate deletion mutations. The numbers in brackets on the left side of the figure indicate the number of identical clones in each sample; the red letters in the top LN40 consensus sequence represent the main mutation hotspots of the LTR, and the underlined regions indicate the locations of the various transcription factor binding sites and the TSS.

FIG S4

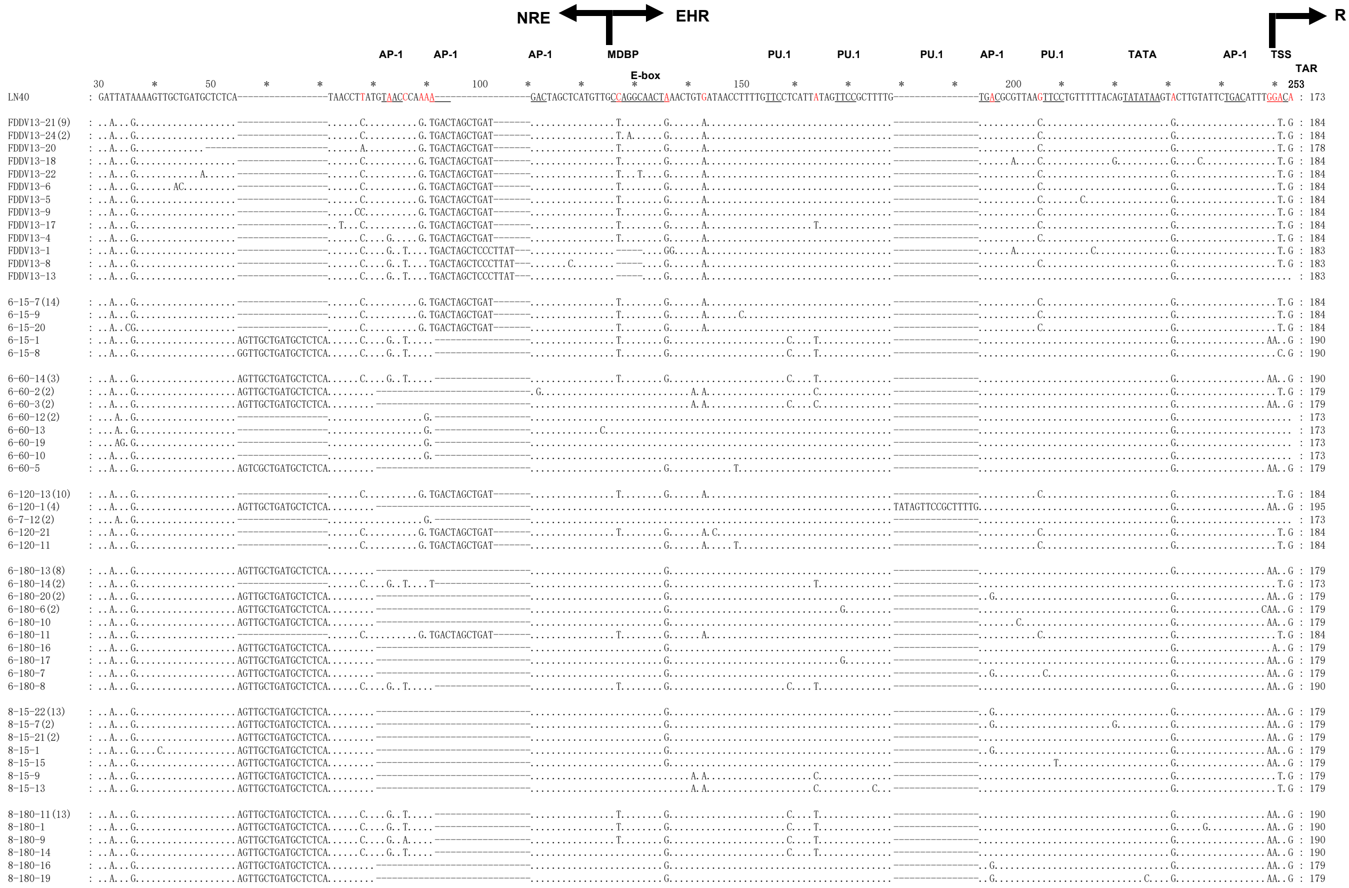

FIG. S4. Sequence comparison of LTRs from FDDV13-infected horses. All LTR sequences of the samples were aligned with the LN40 consensus sequence, which is the reference sequence. Nucleotides that are the same as those in the reference sequence are represented by dots, and nucleotides differing from those in the reference sequence are represented by the corresponding letter symbols; the dashes indicate deletion mutations. The numbers in brackets on the left side of the figure indicate the number of identical clones in each sample; the red letters in the top LN40 consensus sequence represent the main mutation hotspots of the LTR, and the underlined regions indicate the locations of the various transcription factor binding sites and the TSS.
